# Genetic Susceptibility to Type 2 Diabetes and Obesity: Follow-Up of Findings from Genome-Wide Association Studies

**DOI:** 10.1155/2014/769671

**Published:** 2014-02-25

**Authors:** Kevin J. Basile, Matthew E. Johnson, Qianghua Xia, Struan F. A. Grant

**Affiliations:** ^1^Division of Human Genetics, The Children's Hospital of Philadelphia Research Institute, 34th and Civic Center Boulevard, Philadelphia, PA 19104, USA; ^2^Center for Applied Genomics, The Children's Hospital of Philadelphia Research Institute, 34th and Civic Center Boulevard, Philadelphia, PA 19104, USA; ^3^Department of Pediatrics, Perelman School of Medicine, University of Pennsylvania, Philadelphia, PA 19104, USA; ^4^1216F Children's Hospital of Philadelphia Research Institute, 34th and Civic Center Boulevard, Philadelphia, PA 19104, USA

## Abstract

Elucidating the underlying genetic variations influencing various complex diseases is one of the major challenges currently facing clinical genetic research. Although these variations are often difficult to uncover, approaches such as genome-wide association studies (GWASs) have been successful at finding statistically significant associations between specific genomic loci and disease susceptibility. GWAS has been especially successful in elucidating genetic variants that influence type 2 diabetes (T2D) and obesity/body mass index (BMI). Specifically, several GWASs have confirmed that a variant in transcription factor 7-like 2 (*TCF7L2*) confers risk for T2D, while a variant in fat mass and obesity-associated protein (*FTO*) confers risk for obesity/BMI; indeed both of these signals are considered the most statistically associated loci discovered for these respective traits to date. The discovery of these two key loci in this context has been invaluable for providing novel insight into mechanisms of heritability and disease pathogenesis. As follow-up studies of *TCF7L2* and *FTO* have typically lead the way in how to follow up a GWAS discovery, we outline what has been learned from such investigations and how they have implications for the myriad of other loci that have been subsequently reported in this disease context.

## 1. Introduction

Understanding the patterns of heritability associated with the manifestation of specific traits and diseases is one of the fundamental goals of genetic research. However, most of the common traits exhibit complex patterns of heritability that are influenced by altered susceptibility to certain environmental factors resulting from the cumulative contribution of many distinct genetic variants. Due to this complexity, elucidating the genetic causes of these multifactorial diseases has required unbiased, large-scale genome-wide approaches.

These types of approaches became possible as the result of data obtained from the International HapMap Project, which provided a “road map” for genetic variation across the entire genome [1, 2]. This project was able to determine that certain single-nucleotide polymorphisms (SNPs) “traveled” together in heritable blocks. Therefore, haplotypes could be tracked by determining the sequence of a minimal set of SNPs. This knowledge made it possible for the subsequent development of cost-effective, high-throughput genotyping arrays that were capable of accurately determining genome-wide genetic variation in any given patient sample [3–5].

The implementation of this technology led to the birth of genome-wide association study (GWAS) methodologies. These approaches are best suited to identify common variants within the population that yields a modest risk for a specific disease. GWAS has aided in elucidating some of the heritability of various complex diseases by identifying genetic variants associated with diseases such as asthma [6, 7], Parkinson's disease [8, 9], and autism [10]. Indeed, GWAS has been essential in determining the key underlying genetic components associated with type 2 diabetes (T2D).

## 2. Loci Associated with T2D

Prior to the utilization of GWAS approaches, only a handful of genetic events had been shown to be clearly associated with T2D. These loci were identified via candidate gene analyses and family-based linkage studies, which include peroxisome proliferator-activated receptor gamma (*PPARG*), calpain 10 (*CAPN10*), and potassium inwardly rectifying channel, subfamily J, member 11 (*KCNJ11*) [11–13]. However, the advent of GWAS in the mid-2000s has been transformative for gene discovery in this disease, with tens of loci identified to date [14–26] (Table 1).

These loci have been identified and confirmed through several GWAS analyses. Initial studies identified T2D-associated variants in loci harboring genes such as CDK5 regulatory subunit associated protein 1-like 1 (*CDKAL1*), solute carrier family 30 (zinc transporter), member 8 (*SLC30A8*), homeobox hematopoietically expressed (*HHEX*), insulin-like growth factor 2 mRNA-binding protein 2 (*IGF2BP2*), and cyclin-dependent kinase inhibitor 2A/2B (*CDKN2A*/*2B*) [17–19, 23, 26]. Another study using meta-analysis of T2D data found further T2D-associated variants in loci harboring genes including juxtaposed with another zinc finger gene 1 (*JAZF1*), cell division cycle 123, calcium/calmodulin-dependent protein kinase 1D (*CDC123*, *CAMK1D*), tetraspanin 8, leucine-rich repeat-containing G protein-coupled receptor 5 (*TSPAN8*, *LGR5*), thyroid adenoma associated thyroid adenoma associated (*THADA*), ADAM metallopeptidase with thrombospondin type 1 motif, 9 (*ADAMTS9*), and notch gene homolog 2 (*NOTCH2*) [25]. Additionally, a subsequent larger meta-analysis found T2D-associated variants in loci harboring genes such as B-cell cll/lymphoma 11A (*BCL11A*), centaurin, delta-2 (*CENTD2*), coiled-coil-helix-coiled-coil-helix domain 9 (*CHCHD9*), dual-specificity phosphatase 9 (*DUSP9*), high mobility group at-hook 2 (*HMGA2*), hepatocyte nuclear factor-1-alpha (*HNF1A*), potassium voltage gated channel, KQT-like subfamily, member 1 (*KCNQ1*), kruppel-like factor 14 (*KLF14*), protein regulating cytokinesis 1 (*PRC1*), tumor protein p53-inducible nuclear protein 1 (*TP53INP1*), zinc finger BED domain-containing protein 3 (*ZBED3*), and zinc finger, an1-type, domain-containing protein 6 (*ZFAND6*) [22].

T2D loci have also been identified through GWAS of related traits. For example, GWASs of fasting glucose concentrations have discovered a variant in melatonin receptor 1B (*MTNR1B*), which was also subsequently found to be associated with T2D [27–29]. GWAS analysis of fasting glucose concentrations conducted by the meta-analyses of glucose and insulin-related traits consortium (MAGIC) also demonstrated association of adenylate cyclase-5 (*ADCY5*), prospero-related homeobox-1 (*PROX1*), glucokinase (*GCK*), glucokinase regulatory protein (*GCKR*), and diacylglycerol kinase (*DGKB*) transmembrane protein-195 (*TMEM195*) with T2D [14]. Additionally, GWASs for obesity have clearly shown association with fat mass and obesity-associated protein (*FTO*) [30]. Since obesity is known to be a predisposing factor for the development of T2D, it is not surprising that variants in *FTO* have also been found in T2D GWAS [23].

However, the locus that shows the strongest association with T2D in a number of ethnicities occurs in the wnt-signaling pathway member, transcription factor 7-like 2 (*TCF7L2*) [31]. The variant found at this locus is common among the population, occurs in the noncoding region of the gene and results in an approximately 40% increase in T2D risk [31] when considered in isolation. This variant was determined to be consistently and significantly associated with T2D based on a meta-analysis conducted just a year after the initial publication, yielding a *P* value of 5.4 × 10^−140^ [32] and has been confirmed via several T2D GWAS subsequently [17–19, 22]. However, it is still unclear how variation at the *TCF7L2* locus influences the gene product's function and how this altered function drives T2D pathogenesis.

## 3. Missing Heritability of T2D

Despite all of the loci that have been found to be associated with T2D, there are still many more T2D-associated variants that remain to be discovered. Currently, it is thought that the variants that have already been shown to be associated with T2D account for less than 10% of the genetic heritability of the disease. Because GWAS approaches are best suited to identify common variants within the population, it is believed that the remaining variants are rare and likely confer minimally increased risk. These speculations describe a type of genetic heritability that would be similar to that of other complex traits such as height [33]. In order to uncover these missing variants, modifications to the genome-wide approaches used to date are necessary.

For example, many of the initial GWAS analyses were conducted in cohorts of European ancestry. This is due to the fact that there are large and well-categorized collections of this ancestry available for analyses. Additionally, many of the GWAS consortiums and collaborations exist between researchers in Europe and North America. Therefore, GWAS analyses conducted in European populations may miss loci that are more relevant and easier to detect in other ethnicities.

In the case of T2D, it is already clear that variants that confer susceptibility vary across ethnicities. For example, the T2D-associated variant in the *TCF7L2* locus does not occur at a high frequency in Chinese and Japanese populations. Instead, the strongest T2D-associated variant in these populations occurs in *KCNQ1* [21, 24]. In order to further elucidate the genetic landscape of T2D across different ethnicities, several recent GWAS analyses of T2D have been conducted in non-European cohorts. Many of these recent GWAS analyses have been conducted in cohorts of Asian ancestry [34, 35]. Additionally, a recent study has found that sequence variants in *SLC16A11* and *SLC16A13* increase risk of T2D in Mexican populations [36].

Rare variants may also not be detected with a high degree of statistical significance with the samples sizes currently used in GWAS approaches. Meta-analyses and consortiums have increased samples sizes well in excess of 1000 cases and 1000 controls. This has proven sufficient for several “low-hanging fruit” signals; however, the statistical power provided by these large analyses may still be unable to detect rarer variants that may be driving the biggest proportion of the genetic contribution to T2D. Therefore, further collaborations and meta-analyses are necessary to further increase statistical power.

Rare variants may also be missed due to the limitations of current genotyping arrays. For example, current genotyping arrays generally do not provide data for rare genetic variants (<1% frequency) that may well be contributing to T2D risk. Additionally, rare variants may only be loosely in linkage disequilibrium with more common variants at a given locus; indeed, if this is the case, the association signal at these particular loci would be drastically underestimated and may not even achieve statistical significance [37, 38]. Trait associated variants may also be masked by epistatic events; this means that certain variants only contribute to the genetic heritability of T2D in the context of other “modifying” events. In order to move beyond these limitations, high-throughput, whole genome sequencing methods are necessary. However, whole genome sequencing would result in dramatically more data and would require increased analysis and computational power.

Missing heritability of complex diseases may also lie outside of SNPs altogether. Although there is little evidence that copy number variations (CNVs) contribute to a large degree to the genetic heritability of T2D [39], it is possible that rare CNVs may contribute to certain cases of the disease and would not be detected via current genotyping arrays. Other variations in genomic structure, such as inversions, may also be difficult to detect using genotyping arrays or next-generation sequencing methods. Additionally, heritability of T2D may be influenced by epigenetic factors such as DNA methylation and histone modification. Genome-wide analyses suited to detect these modifications would need to be conducted in order to adequately determine their influence. However, other dynamic changes in the epigenome and possible tissue-specific epigenetic modifications may further complicate analyses aiming to identify heritability mediated by these mechanisms.

## 4. T2D Loci: Insight into the Disease

While there is still quite a bit of work to be done to determine the complex genetic heritability of T2D, the variants already identified by GWAS have successfully provided insight into the biology of the disease. For example, genetic studies have shown a strong correlation between low birth weight and the development of diabetes [40–42]. Interestingly, many of the loci that have been shown to be associated with T2D have been implicated in birth weight determination [43–47]. Similarly, it has also been shown that the T2D-associated locus in *HHEX* is associated with increased childhood body mass index (BMI) [48]. This means that the influence of these T2D risk loci may exert their effects early on in life.

Additionally, manipulating the genes nearest to the T2D-associated variants may provide insight into possible therapeutic strategies. These strategies could be designed either to delay the onset of T2D or to relieve symptoms associated with the disease. In fact, some of the established T2D-associated genes are already known drug targets, for example, *PPARG* and thiazolidinediones (TZDs), a drug class used to treat T2D by activating PPARs [11] and *KCNJ11* and sulfonylureas [12, 49].

Although GWAS has provided a degree of insight into disease biology by identifying variants in specific genetic loci, the exact function of these variants has rarely been elucidated. One of the main reasons why the functional ramifications of GWAS associated loci are not determined is the fact that the signal obtained through GWAS does not clearly identify the causal variant. As mentioned earlier, the genotyping arrays used in GWAS determine a person's haplotype using a minimal set of SNPs. This means that the sequence of other SNPs in a linkage disequilibrium block can be inferred, or “imputed,” based on the determination of a specific “tag” SNP. For this reason, the association signal identified through GWAS only serves to limit the search to a number of possible causal variants within a narrow genomic region. Current inroads are being made to identify the causal variants from the majority of GWAS analyses.

## 5. T2D Risk Predictions

Loci that are clearly associated with T2D have also been tested for their ability to act as cumulative risk predictors. Unfortunately, initial sets of T2D-associated variants failed to add significant predictive efficacy. For example, one study assessed the predictive effect of 18 SNPs in genes associated with T2D [50]. These SNPs included *NOTCH2* (rs10923931), *BCL11A* (rs10490072), *THADA* (rs7578597), *IGF2BP2* (rs1470579), *PPARG* (rs1801282), *ADAMTS9* (rs4607103), *CDKAL1* (rs7754840), vascular endothelial growth factor A (*VEGFA*) (rs9472138), *JAZF1* (rs864745), *SLC30A8* (rs13266634), *CDKN2A*, *CDKN2B* (rs10811661), *HHEX* (rs1111875), *CDC123*, *CAMK1D* (rs12779790), *TCF7L2* (rs7903146), *KCNJ11* (rs5219), insulin (*INS*) (rs689), dermcidin (*DCD*) (rs1153188), and *TSPAN8*, *LGR5* (rs7961581) [50]. This study found that the relative risk of diabetes increased by 12% per risk allele [50]. Additionally, this study found that the risk of T2D was increased by a factor of 2.6 between individuals with the lowest genotype risk scores and individuals with the highest genotype risk scores [50]. However, the addition of this genotype score did not improve risk discrimination when familial diabetes or other documented clinical risk factors were considered [50].

Another study that was published concurrently with the previously described study assessed the predictive effect of 16 SNPs in genes associated with T2D [51]. These SNPs included *TCF7L2* (rs7903146), *KCNJ11* (rs5219), *PPARG* (rs1801282), *CDKAL1* (rs7754840), *IGF2BP2* (rs4402960), *CDKN2A*, *CDKN2B* (rs10811661), *FTO* (rs9939609), *HHEX* (rs1111875), *SLC30A8* (rs13266634), wolfram syndrome 1 (*WFS1*) (rs10010131), *JAZF1* (rs864745), *CDC123*, *CAMK1D* (rs12779790), *TSPAN8*, *LGR5* (rs7961581), *THADA* (rs7578597), *ADAMTS9* (rs4607103), and *NOTCH2* (rs10923931) [51]. Eleven of these 16 loci were found to be significantly associated with the risk of T2D, including loci in *TCF7L2*, *PPARG*, *FTO*, *KCNJ11*, *NOTCH2*, *WFS1*, *CDKAL1*, *IGF2BP2*, *SLC20A8*, *JAZF1*, and *HHEX* [51]. However, similar to the previously described study, the inclusion of these loci only slightly improved prediction of future T2D compared with the inclusion of clinical risk factors alone [51].

However, as more variants were found to be associated with T2D, the efficacy of risk prediction for the disease improved. For example, one recent study found that a set of 40 SNPs associated with T2D could predict the risk of younger people for T2D but not for older people [52]. Another study published around the same time found that 34 confirmed that T2D loci were sufficient to predict increased risk for developing the disease [53]. A more recent study has also demonstrated that T2D risk could be predicted using a set of 46 known associated variants [54]. However, as mentioned in a previous section, much of the heritability of T2D is currently uncharacterized; as such, although up to 65 common T2D susceptibility loci have been formally established with genome-wide significance in populations of European descent [22, 55], risk prediction for T2D is far from optimal.

## 6. Relationship of T2D and Other Diseases

Although cumulative risk prediction of T2D using known disease-associated loci needs further improvement, the vast number of loci associated with the disease and the even greater number of risk-conferring loci that remain to be identified may reveal interesting genetic interactions in specific cellular signaling pathways. For instance, one of the main variants associated with T2D risk occurs within the genetic region of a known cancer associated locus, namely, *TCF7L2* (formerly known as* TCF4*), where mutations in this genehave been strongly associated with colorectal cancer risk specifically [56, 57]. Additionally, genomic sequencing of colorectal adenocarcinomas has recently identified a recurrent vesicle transport through interaction with t-SNAREs 1A (*VTI1A*)-*TCF7L2* gene fusion [58]. Supporting the relationship between T2D and cancer, many of the risk loci associated with T2D have been strongly detected in separate GWAS analyses of prostate cancer, including *THADA*, *JAZF1*, and *HNF1B* [15, 25, 59, 60]. Thus, some of the T2D-associated genes also appear to be key players in cancer pathogenesis.

Although the association between T2D loci and cancer-related genes is intriguing, the importance of this relationship is still far from understanding. However, it should be noted that the risk-conferring allele of several T2D loci has actually been shown to protect against prostate cancer [59]. Alternatively, the non-risk-conferring allele of these T2D loci is strongly associated in prostate cancer patients. Therefore, there may be a specific yin and yang relationship between cancer and T2D, with one hypothesis being that cancer is the result of too much cell proliferation and T2D is the result of insufficient proliferation of cells, that is, pancreatic islets.

T2D also shares commonalities with more related diseases, such as mature onset diabetes of the young (MODY). Although MODY and T2D are both classified as non-insulin-dependent diabetes mellitus (NIDDM), the genetic heritability of MODY is relatively monogenic versus the complex, polygenic predisposition that is attributed to the heritability of T2D. Currently, there are thirteen distinct loci that have been confirmed to be causal for MODY: hepatocyte nuclear factor 4 (*HNF4A*), *GCK*, *HNF1A*, pancreatic and duodenal homeobox 1 (*PDX1*), *HNF1B*, neurogenic differentiation 1 (*NEUROD1*), kruppel-like factor 11 (*KLF11*), carboxyl ester lipase (*CEL*), paired box 4 (*PAX4*), *INS*, B lymphoid tyrosine kinase (*BLK*), potassium inwardly rectifying channel, subfamily J, member 11 (*KCNJ11*), and ATP-binding cassette, subfamily C, member 8 (*ABCC8*) [61–65]. Interestingly, many of the genes mutated in MODY have been found to be associated with T2D through GWAS analyses. For example, two independent SNPs in the *HNF4A* locus (rs6017317 and rs4812829) were found to associate with T2D in individuals of Asian ancestry [34, 35]. Additionally, SNPs in the genomic loci of *HNF1A* (rs7957197) and *HNF1B* (rs4430796) were found to be associated with T2D in individuals of European ancestry [22]. A study of T2D in individuals of Asian ancestry also found a SNP in *PAX4* (rs6467136) that showed strong association in cases versus controls [34].

Since the genetic mutations that cause MODY occur in genes associated with glucose homeostasis, it is not entirely surprising that some of these genes may also be impacted in cases of T2D. Prior to GWAS analyses, previous studies found evidence to suggest that missense mutations in the *PDX1* gene result in enhanced predisposition to developing T2D, especially at a young age [66, 67]. Additionally, one study found that a mutation in *HNF1A* may be linked to T2D in the Canadian Oji-Cree Indians [68]. These findings suggest that predisposition to T2D may be enhanced by polygenic alterations in MODY-associated genes. Additionally, alterations in MODY-associated genes may accelerate the age of onset of T2D in certain patients.

Although many MODY genes have also been implicated in T2D, the relationship between genetic loci associated with T2D and genetic loci associated with type 1 diabetes (T1D) is virtually nonexistent. Both T2D and T1D are associated with an inability to maintain appropriate blood glucose levels as a result of mitigated insulin production/sensitivity. However, lack of insulin production associated with T1D is caused by an autoimmune disorder in which the body attacks and destroys the insulin-producing beta cells of the pancreas. Because T1D is the result of an autoimmune disorder, clinical diagnosis can be confirmed via detection of distinct autoantibodies in the blood. Therefore, despite having similar symptoms, T2D and T1D are two distinct diseases.

Confirming this dissimilarity, GWAS analyses have repeatedly demonstrated that loci associated with T2D are almost entirely distinct from loci associated with T1D [23, 69–74]. For example, one major GWAS of T1D found that over 40 loci influenced the risk of developing the disease [69]. However, only one of these loci (GLIS family zing finger 3 (*GLIS3*)-rs7020673) was found in a region near a T2D-associated gene (*GLIS3*-rs7041847) [34, 69], albeit the fact that it has still to be fully resolved if this signal was in fact driven by the presence of undiagnosed late onset autoimmune diabetics in the cohort studied [75]. Additionally, a study found that 10 distinct T2D-associated loci showed no significant association in cases of T1D [76]. Therefore, although both diseases are influenced by polygenic predisposition, the findings of these and related studies confirm the lack of a relationship between genetic loci associated with T2D and genetic loci associated with T1D.

Genome-wide screening of patients with T2D has clearly identified unique genetic factors that contribute to the development of the disease. However, many factors remain to be identified in order to accurately describe its complex genetic heritability. Careful attention needs to be given to the relationship of genetic loci associated with T2D and genetic loci associated with other diseases, such as prostate cancer and MODY. Comparing the characteristics of these diseases and the genetic alterations they seem to share may provide additional clarity in discerning novel clues about T2D pathogenesis.

## 7. Proposed Mechanisms of *TCF7L2* in T2D

As mentioned above, it is generally accepted that the most strongly associated locus with T2D is *TCF7L2*. The *TCF7L2* gene encodes a high mobility group (HMG) box-containing transcription factor, which is the effector of the canonical Wnt signaling pathway that plays a key role in embryonic development [77, 78]. Prior to its association with T2D, *TCF7L2* was identified as a cancer gene. TCF7L2 protein regulates the expression of several genes including cyclin D1 and c-myc, which control the G1 to S phase transition in the cell cycle [79, 80].

The rs7903146 SNP in *TCF7L2* is widely considered to be causal through studies in multiple ethnicities [81, 82] and with Bayesian modeling [83]. Interestingly, the risk T allele of rs7903146 is common in European and African populations but not in the Han Chinese population, which may explain the inconsistent results of replication studies using Chinese populations [84–89].

Like most common variants associated with T2D uncovered by GWAS, rs7903146 is located within a noncoding region, that is, intron 3. However, the underlying mechanism of how the T allele increases risk for T2D remains unknown. Several hypotheses have been proposed to explain the causal role of this SNP. These hypotheses are based on preliminary data such as allele-specific expression level, splicing variants, and chromatin structure [90]. Some studies suggest that *TCF7L2* mRNA levels increase with the number of T alleles in human pancreatic islets, and overexpression of *TCF7L2* in human islets causes impaired insulin secretion [91, 92]. However, other studies found that *TCF7L2* expression levels were not altered by genotype or associated with either insulin sensitivity or BMI [93]. These contradictory results could be due to different experimental approaches such as real-time PCR for mRNA expression versus immunostaining for protein levels. Additionally, results from studies using small sample sizes may not have had sufficient statistical power to achieve significance [94].

Although the *TCF7L2* gene has been reported to undergo extensive alternative splicing, there is currently no conclusive evidence that alternative splicing of *TCF7L2* is associated with variations in genotype. A larger sample population may be required to reveal any allele-specific effects on splicing pattern [95]. However, one elegant study using formaldehyde-assisted isolation of DNA analyzed with high-throughput sequencing (FAIRE-seq) found that the risk T allele of rs7903146 was more abundant than the nonrisk C allele in the open chromatin fraction, suggesting the role of the allele-specific changes in transcriptional activity, promoter usage, or splicing [90].

Other studies have investigated further hypotheses related to the role of *TCF7L2* in T2D. These studies have sought to clarify the role of *TCF7L2* in a variety of organs/tissues including islets, gut, adipose, or liver. However, most studies focused on the pancreatic islet [96]. Depletion of *TCF7L2* using small interfering RNA in human islets resulted in decreased *β*-cell proliferation and increased *β*-cell apoptosis. Additionally, glucose-stimulated insulin secretion (GSIS) was impaired by loss of *TCF7L2* in both mouse and human islets, whereas overexpression of *TCF7L2* in islets was resistant to glucose and cytokine-induced apoptosis and impaired function [97]. Dominant-negative *TCF7L2* repressed the proliferation of rat INS-1 cells, suggesting the role of *TCF7L2* in maintaining beta cell mass [98].

*TCF7L2* may also play a role in gastrointestinal tissues. Knockout of *TCF7L2* was embryonic lethal in mice due to defects in proliferation of the crypt stem cells of the small intestine [99]. *TCF7L2* may also play a critical role in glucose homeostasis through the hormone glucagon-like peptide 1 (GLP-1) produced in the enteroendocrine L cells in the small intestine [94]. *TCF7L2* binds to the promoter region of proglucagon gene and controls its transcriptional activity in the intestinal GLUTag cell line, where the dominant-negative mutant of *TCF7L2* abolishes proglucagon mRNA levels [100].

Interestingly, recent studies revealed the important role of *TCF7L2* in metabolism using a conditional knockout mouse model [101–103]. Deletion of *TCF7L2* from *β*-cells revealed no effect of embryonic development of the endocrine pancreas, *β*-cell proliferation, and expression of relevant *β*-cell genes. However, hepatic lipid metabolism was impaired by liver-specific deletion of *TCF7L2* [101]. This suggests that *TCF7L2* may function in metabolic adaptation after birth, but not during embryonic development. Further analyses revealed that the expression of several key genes involved in glycogen metabolism, such as glycogen synthase 2 (*GYS2*), and gluconeogenesis, such as phosphoenolpyruvate carboxykinase 1 (*PCK1*) and glucose-6-phosphate, catalytic subunit (*G6PC*), was significantly reduced in *TCF7L2* knockout newborn livers when compared with wild-type (WT) littermates [101].

Other studies have found that the expression of *TCF7L2* was decreased in human subcutaneous adipose tissue from patients with T2D, which further suggests that *TCF7L2* plays a role in various metabolic tissues [104]. Furthermore, studies have shown tissue-specific alternative splicing of *TCF7L2* [105–107]. However, functional studies of *TCF7L2* in these other metabolic tissues are still lacking.

## 8. *TCF7L2* and Drug Response

Since *TCF7L2* variants have been shown to be strongly associated with T2D, it is reasonable to propose that *TCF7L2* genotypes may influence drug response. Indeed, clinical studies have shown that *TCF7L2* variants alter therapeutic response to sulfonylureas but not metformin in patients with T2D. The carriers of the risk alleles at rs12255372 and rs7903146 have a higher rate for sulfonylurea treatment failure [108, 109]. These results may be explained by the different mechanisms of these two drugs. Sulfonylureas increase insulin release from the *β*-cells in the pancreas, while metformin suppresses glucose production by the liver (hepatic gluconeogenesis) by improving the action of insulin [110]. These data suggest that therapeutic efficacy in T2D may be partially determined by genotypes, providing an insight for personalized therapeutics.

## 9. *TCF7L2* Chromatin Binding

Chromatin immunoprecipitation sequencing (ChIP-seq) experiments from livers of mice identified many genes involved in metabolic pathways including Wnt target genes such as Axin2 and Sp5. Gene set enrichment analysis demonstrated that these genes were involved in multiple metabolic processes, including lipid metabolism, carbohydrate, steroid, and ketone, suggesting an essential role of *TCF7L2* in metabolic diseases [101]. Additionally, pathway analysis of *TCF7L2* ChIP-seq data from the colorectal cancer cell line HCT116 demonstrated that the most significant pathways were associated with T2D and cancer [111]. These findings reveal a possible central role of *TCF7L2* in metabolic disease.

## 10. *TCF7L2* in Other Disease Contexts

Recent studies have shown that *TCF7L2* may play a role in other diseases besides T2D and colorectal cancer. It is has been reported that the TCF7L2 protein binds to the 8q24 genomic locus, which regulates c-myc oncogene associated with multiple cancers [112]. A recent GWA study from Hispanic and non-Hispanic white (NHW) women found that four *TCF7L2* SNPs (including rs7903146) were significantly associated with breast cancer [113]. Additionally, *TCF7L2* rs7903146 has been identified as a susceptibility locus for latent autoimmune diabetes of adults (LADA) in Europeans [114]. Several studies have also demonstrated that the genetic variants of *TCF7L2* that drive T2D risk also increase susceptibility to cystic fibrosis-related diabetes (CFRD) [115–117]. Interestingly, meta-analysis showed that maternal *TCF7L2* genotype was associated with increased offspring birth weight. This increase may be caused by impaired maternal insulin secretion [116].

## 11. *FTO* in Obesity

As mentioned earlier, the variant most strongly associated with obesity/BMI is found in the *FTO* locus. This SNP was originally identified through T2D GWAS, which determined an association with the rs9939609 variant in the intronic region of this gene [30]. Since it was well known that T2D and BMI phenotypes are associated with each other, the original study proposed adjusting the *FTO* locus for BMI, which abolished the T2D association. This indicated that the primary effect of the SNP variant was mainly due to BMI and not T2D. Around the same time as this study was published, another group also found that the *FTO* locus was associated with susceptibility to obesity [118]. In this study, *FTO* association was found during the examination of 48 SNPs which were used as markers of population stratification in a genomic region that was thought to be intergenic. However, a SNP in this region mapped to the *FTO* locus and was shown to be strongly associated with extreme childhood obesity [118]. These two studies indicate that properly defining disease phenotype is crucial for GWAS to determine the association of specific SNPs with the disease of interest. Additionally, these studies indicate that using BMI as a maker of obesity can introduce systematic errors of over/underestimation of adiposity in Asian, Africa, and Hispanic racial groups, which can make selecting obesity cases and population controls in these racial groups challenging [119, 120].

## 12. Further Studies Show *FTO* Association with Obesity

Recently, large-scale obesity/BMI GWAS approaches that had typically been restricted to European populations have been extended to Asian populations. For example, two large-scale GWAS in Asian populations using approximately 28,000 individuals was the largest GWAS of obesity/BMI in individuals of non-European descent thus far [121, 122]. Interestingly, a GWAS which included East Asians (Chinese, Korean, and Indonesian) including 27,715 obesity patient samples was able to replicate the previously identified *FTO* locus in European population in East Asian populations [122]. Another study of 26,620 obese Japanese patients also replicated previous findings of the *FTO* locus [121].

Recently, investigators have also sought to conduct GWAS of obesity/BMI in diverse African populations. However, conducting GWAS in these populations has proven to be problematic; for example, initial analyses in these populations could not replicate the association of the *FTO* previously found in European populations due to the small degree of linkage disequilibrium in the African cohorts [119]. Further analyses of African American children also could not find association of the *FTO* SNP with obesity [72, 123, 124]. However, increasing sample size via a large-scale meta-analysis was able to replicate the *FTO* association in African populations [125].

In order to overcome challenges of SNP association in African populations, a more refined mapping of SNP association of *FTO* was performed in populations of African Americans (*n* = 968) and West Africans (*n* = 517) covering 262 tag SNPs across the entire *FTO* gene [126]. The selection of these SNPs allowed the authors to determine significant association within the *FTO* intronic regions, which replicated what had been previously reported in European studies [126]. In a recent SNP association study, 44 known BMI-associated SNPs or tagged SNPs in genes associated with appetite regulation were tested for association with obesity in black South African adolescents. The only SNP that was determined to be significantly associated with BMI in this study was the *FTO* variant rs17817449 [127]. This suggests that the SNP in *FTO* could be utilized as a potential genetic marker of obesity risk in African populations.

## 13. Determining the Function of *FTO* in Obesity

Although the variant found in the *FTO* locus clearly showed association with BMI, the function of this gene in relation to obesity was unclear at the time. *FTO* had been previously cloned after identification of a fused-toe mutant (*FTO*) mouse whose phenotype results from a 1.6 Mb deletion of six genes including *FTO* [128]. The *FTO* gene consists of nine exons spanning ~400 kb on chromosome 16 in humans and is present in all vertebrates [129–131]. By utilizing a recombinant murine *FTO* gene, it was shown that *FTO* catalyzes the demethylation of 3-methylthymine in single-stranded DNA, which suggests a potential role in nucleic acid demethylation [129]. Additional studies also found that *FTO* localizes to the nucleus, and *FTO* mRNA is abundantly expressed in the brain [129]. Specifically, the abundant expression of *FTO* mRNA was found to occur in the hypothalamic nuclei of the brain, which have been determined to be involved in governing energy balance. Additionally, *FTO* expression levels in the arcuate nucleus of the mediobasal hypothalamus were shown to be regulated by feeding and fasting in the mouse brain [129]. However, the potential influence of *FTO* expression on the demethylation of single-stranded DNA and mRNA in the brain is still unclear.

Individuals homozygous for the *FTO* high risk (A) allele were found to weigh approximately three kilograms more than individuals with the low risk (T) allele [132]. Additionally, children homozygous for the low risk (T) allele of *FTO* were shown to eat significantly less than heterozygotes (*P* = 0.03) or AA homozygotes (*P* = 0.02) suggesting that the T allele is protective against overeating by promoting neurological signals for satiety [133, 134]. Mouse models lacking *FTO* gene function or expression exhibit increased energy expenditure and lean phenotype suggesting that loss of function protects against obesity in mice [132, 135, 136]. The mouse model that ubiquitously overexpresses *FTO* showed increases in body and fat mass, which were independent of diet, suggesting that this increase was primarily due to an increased rate of food intake [132]. Taken together, these studies suggest that the influence of *FTO* on obesity status may be operating in a neuropsychiatric fashion through the hypothalamus leading to loss of appetite control.

## 14. *FTO* in Childhood Obesity

The original focus of most published obesity/BMI GWAS was primarily on the association with adult BMI. However, findings in adult obesity/BMI GWAS of common variants in or near *FTO* revealed that these variants were also associated with childhood BMI and childhood obesity [30, 72, 137, 138]. Expanding these association studies to focus on childhood BMI and obesity has provided insights into their mechanisms of action during childhood development through adulthood. In fact, studies that examined the role of *FTO* in childhood obesity determined that the association observed in children was almost identical to that of adults [72, 137, 138]. In a life course study of *FTO* association with BMI in the MRC National Survey of Health and Development, it was also demonstrated that the high risk (A) allele of the *FTO* susceptibility locus was positively associated with BMI, and this association strengthened between ages 2 and 20 years [139]. These data suggest that variants of *FTO* may be primarily associated with early onset obesity leading to greater early infancy weight gain contributing to the adult onset obesity risk, and thus greater T2D risk, seen in adult association studies.

## 15. Conclusion

Genome-wide approaches, such as GWAS, have been invaluable for elucidating mechanisms of heritability associated with various complex diseases. These approaches have been especially successful in discovering key genetic variants associated with T2D and obesity/BMI. Researchers should continue utilizing and modifying these types of genome-wide approaches in order to discover other genetic susceptibility loci and further clarify the missing heritability associated with these complex diseases. Elucidating the underlying genetic variations influencing these complex diseases will be crucial for identifying specific targets for future therapeutic interventions employing techniques that have already been leveraged for *TCF7L2* and *FTO*.

## Figures and Tables

**Table 1:** Type 2 diabetes loci established to date.

Gene symbol	Full gene name	References
*ADAMTS9 *	Thyroid adenoma associated (*THADA*), ADAM metallopeptidase with thrombospondin type 1 motif, 9	[25]
*ADCY5 *	Adenylate cyclase 5 (*ADCY5*)	[14, 45]
*ADRA2A *	Alpha-2a-adrenergic receptor	[14]
*AP3S2 *	Adaptor-related protein complex 3, sigma 2 subunit	[35]
*BCL11A *	B-cell cll/lymphoma 11A	[22]
*C2CD4B *	C2 calcium-dependent domain-containing protein	[14]
*CCNL1 *	Cyclin L1	[14, 45]
*CDC123-CAMK1D *	Cell division cycle 123 homolog/calcium/calmodulin-dependent protein kinase ID	[25]
*CDKAL1 *	CDK5 regulatory subunit associated protein 1-like 1	[17–19, 23, 26]
*CDKN2A/B *	Cyclin-dependent kinase inhibitor 2A/B	[17–19, 23, 26]
*CENTD2 *	Centaurin, delta-2	[22]
*CHCHD9 *	Coiled-coil-helix-coiled-coil-helix domain 9	[22]
*CMIP *	c-Maf inducing protein	[34]
*CRY2 *	Cryptochrome 2	[14]
*DUSP9 *	Dual-specificity phosphatase 9	[22]
*FADS1 *	Fatty acid desaturase 1	[14]
*FITM2 *	Fat storage-inducing transmembrane protein 2	[34]
*GCC1 *	GRIP and coiled-coil domain containing 1	[34]
*GCK *	Glucokinase	[14]
*GCKR *	Glucokinase regulatory protein	[14]
*GLIS3 *	Glis family zinc finger protein 3	[14, 34]
*GRB14 *	Growth factor receptor-bound protein 14	[35]
*HHEX *	Homeobox hematopoietically expressed	[17–19, 23, 26]
*HMG20A *	High mobility group 20A	[35]
*HMGA2 *	High mobility group at-hook 2	[22]
*HNF1A *	Hepatocyte nuclear factor-1-alpha	[22]
*HNF1B *	HNF1 homeobox B	[15]
*HNF4A *	Hepatocyte nuclear factor-4-alpha	[34, 35]
*IGF1 *	Insulin-like growth factor 1	[14]
*IGF2BP2 *	Insulin-like growth factor 2 mRNA-binding protein 2	[17–19, 23, 26]
*IRS1 *	Insulin receptor substrate 1	[16]
*JAZF1 *	Juxtaposed with another zinc finger gene 1	[25]
*KCNK16 *	Potassium channel, subfamily K, member 16	[34]
*KCNQ1 *	Potassium voltage gated channel, KQT-like subfamily, member 1	[21, 22, 24]
*KLF14 *	Kruppel-like factor 14	[22]
*MADD *	Map kinase-activating death domain	[14]
*MAEA *	Macrophage erythroblast attacher	[34]
*MTNR1B *	Melatonin receptor 1B gene	[27–29]
*NOTCH2 *	neurogenic locus notch homolog protein 2	[25]
*PAX4 *	paired box 4	[34]
*PEPD *	peptidase D	[34]
*PPARG *	peroxisomal proliferator-activated receptor-g	[11]
*PRC1 *	protein regulating cytokinesis 1	[22]
*PROX1 *	prospero-related homeobox 1	[14]
*PSMD6 *	proteasome (prosome, macropain) 26S subunit, non-ATPase, 6	[34]
*R3HDML *	R3H domain containing-like	[34]
*SLC16A11 *	solute carrier family 16, member 11	[36]
*SLC16A13 *	solute carrier family 16, member 13	[36]
*SLC2A2 *	solute carrier family 2, member 2	[14]
*SLC30A8 *	solute carrier family 30, member 8	[17–19, 23, 26]
*ST6GAL1 *	ST6 beta-galactosamide alpha-2,6-sialyltransferase 1	[35]
*TCF7L2 *	transcription factor 7-like 2	[31]
*TMEM195 *	diacylglycerol kinase (DGKB)-transmembrane protein 195	[14]
*TP53INP1 *	Tumor protein p53-inducible nuclear protein 1	[22]
*TSPAN8-LGR5 *	tetraspanin 8/leucine-rich repeat containing G protein-coupled receptor 5	[25]
*VPS26A *	Vacuolar protein sorting 26 homolog A	[35]
*WWOX *	WW domain containing oxidoreductase	[34]
*ZBED3 *	zinc finger BED domain-containing protein 3	[22]
*ZFAND3 *	zinc finger, AN1-type domain 3	[34]
*ZFAND6 *	zinc finger, AN1-type, domain-containing protein 6	[22]
Intragenic		[17–19, 23, 26]

Gene symbol, gene name, and references are provided for each locus.
